# The triple helix of clinical, research, and education missions in academic health centers: A qualitative study of diverse stakeholder perspectives

**DOI:** 10.1002/lrh2.10250

**Published:** 2020-10-17

**Authors:** Jed D. Gonzalo, Michael Dekhtyar, Kelly J. Caverzagie, Barbara K. Grant, Steven K. Herrine, Abraham M. Nussbaum, Darlene Tad‐y, Earla White, Daniel R. Wolpaw

**Affiliations:** ^1^ Department of Medicine Pennsylvania State University College of Medicine Hershey Pennsylvania USA; ^2^ Medical Education Outcomes, American Medical Association Chicago Illinois USA; ^3^ Office of Health Professions Education and Division of General Medicine‐Academic University of Nebraska College of Medicine Omaha Nebraska USA; ^4^ Department of Medicine Sidney Kimmel Medical College Philadelphia Pennsylvania USA; ^5^ Department of Psychiatry University of Colorado School of Medicine Aurora Colorado USA; ^6^ Medicine‐Hospital Medicine University of Colorado School of Medicine Aurora Colorado USA; ^7^ Chair of the Undergraduate Medical Education Department A.T. Still University School of Osteopathic Medicine in Arizona Mesa Arizona USA

**Keywords:** academic health centers, academic medicine, coproduction, health systems science, learning health system

## Abstract

**Introduction:**

Academic health centers are poised to improve health through their clinical, education, and research missions. However, these missions often operate in silos. The authors explored stakeholder perspectives at diverse institutions to understand challenges and identify alignment strategies.

**Methods:**

Authors used an exploratory qualitative design and thematic analysis approach with data obtained from electronic surveys sent to participants at five U.S. academic health centers (2017‐18), with four different types of medical school/health system partnerships. Participants included educators, researchers, system leaders, administrators, clinical providers, resident/fellow physicians, and students. Investigators coded data using constant comparative analysis, met regularly to reconcile uncertainties, and collapsed/combined categories.

**Results:**

Of 175 participants invited, 113 completed the survey (65%). Three results categories were identified. First, five higher‐order themes emerged related to aligning missions, including (a) shared vision and strategies, (b) alignment of strategy with community needs, (c) tension of economic drivers, (d) coproduction of knowledge, and (e) unifying set of concepts spanning all missions. Second, strategies for each mission were identified, including education (new competencies, instructional methods, recruitment), research (shifting agenda, developing partnerships, operations), and clinical operations (delivery models, focus on patient factors/needs, value‐based care, well‐being). Lastly, strategies for integrating each dyadic mission pair, including research‐education, clinical operations education, and research‐clinical operations, were identified.

**Conclusions:**

Academic health centers are at a crossroads in regard to identity and alignment across the tripartite missions. The study's results provide pragmatic strategies to advance the tripartite missions and lead necessary change for improved patient health.

## 1 INTRODUCTION

Health systems are striving toward the Quadruple Aim of improving patient experience, advancing population health, controlling costs of care, and securing clinician well‐being.^1^, ^2^ Achieving these goals will depend on how effectively dynamic and robust principles of system learning and continuous improvement are embedded into processes, structures, and mental models.^3^, ^4^, ^5^ Academic health centers (AHCs) bring an added degree of complexity to this challenge, adding core missions of research and education to the aspirational care outcomes framed by the Institute for Healthcare Improvement (IHI).^6^ Defined as “a constellation of functions and organizations committed to improving the health of patients and populations through the integration of their roles in research, education, and patient care,” AHCs should be well‐positioned to take the lead in improving health and transforming systems of care if their missions are integrated and aligned.^6^, ^7^ Unfortunately, careful analysis often reveals silos rather than synergy.^8^ Mission alignment within an AHC depends on utilizing management, finances, governance, and strategy in the context of a flexible, dynamic work process, and a shared vision.^8^, ^9^

Several forces have challenged the viability and cohesion of AHC tripartite missions including new economic models, particularly the costs associated with education and research, decreased governmental support, and changing health policy.^9^, ^10^, ^11^, ^12^, ^13^ Clinical care is adapting to a stuttering movement from fee‐for‐service to value‐based care, while attempting to respond to the rapid evolution of bioinformatics, technology, and consumerism. Research programs are challenged to fund promising investigators and important scholarship. Health professions education is challenged to reimagine learning agendas to prepare future clinicians to meet patient and system needs.^14^ Academic health centers experience enormous short‐term pressures to remain viable while navigating new and evolving healthcare landscapes that often overshadow the long‐term goal of aligning missions. Collectively, the tripartite missions are inter‐related and require more than independent adaptations to align with evolving needs.^15^ System leaders have reported significant variability in the degree to which medical education and clinical and basic science research are shared within AHCs.^9^ At the same time, although leaders believe alignment is critical, 75% report not knowing how to implement strategies to achieve this.^8^ Limited work has explored the pragmatic translation of alignment into actionable strategies.^9^, ^16^

## 2 RESEARCH AIM

We used qualitative methods of data, obtained from surveys completed by diverse stakeholders, to explore the current AHC landscape with the goal of identifying barriers and opportunities for productive tripartite mission alignment. Our study design allowed a holistic synthesis of both conceptual and pragmatic levels of this research question, both of which are critical for generalizable knowledge. The goal of this study was to explore opportunities to reconceptualize how the tripartite missions could be better aligned and improve health for patients and populations.

## 3 METHODS

### 3.1 Study design

We performed an exploratory qualitative study using thematic analysis of data obtained from a survey with open‐ended questions administered to several stakeholder groups.^17^, ^18^ Institutional review boards at the University of Nebraska Medical Center, Thomas Jefferson University Hospital/Sidney Kimmel Medical College, University of Colorado School of Medicine, A.T. Still University School of Osteopathic Medicine in Arizona, and Penn State College of Medicine (PSCOM) approved this study.

### 3.2 Academic health centers

We explored diverse perspectives about the education, research, and clinical missions of U.S. medical schools and their partnering health system(s). We first categorized U.S. medical schools based on their affiliation(s) with hospitals and organizational structure. Using existing definitions from the Association of American Medical Colleges, Chartis Consulting Group, and Association for Academic Health Centers, we defined an AHC as a hospital affiliated with a medical school, and “integrated” as “being under common ownership with a College of Medicine (COM), having the majority of medical school department chairperson as the hospital chiefs of service, or having the chairperson responsible for appointing the hospital chiefs of service.”^9^, ^19^, ^20^ We identified four categories of medical schools (Data S1) for this work, and identified at least one school from each (Table 1).

**TABLE 1:** Demographics of respondents from each participating medical school and/or health system

	Respondent role categories and number of respondents							
U.S. Medical School and Academic Medical Center Affiliation Category and Participating School	Educator	Researcher	System Leader	Administrator	Clinician	Resident/Fellow	Student	Total
Category 1: Integrated AHC with COM in a public comprehensive/health science university *Penn State College of Medicine/Penn State Health*	3	3	4	2	4	4	4	24
Category 2: Integrated AHC with COM in a private comprehensive/health science university *Sidney Kimmel Medical College at Thomas Jefferson U./Thomas Jefferson U. Hospital*	3	5	5	2	4	3	2	24
Category 3: COM in a public comprehensive or health science university with affiliation agreement (and not under common ownership) with ≥1 AHCs that sponsor/significantly participate in UME and GME *U. of Nebraska School of Medicine/U. of Nebraska Medical Center* *U. of Colorado School of Medicine/U. of Colorado Hospital*	8	3	5	7	3	5	2	33
Category 4: COM in a private comprehensive or health science university with affiliation agreement (and not under common ownership) with ≥1 AHCs that sponsor/significantly participate in UME and GME *A.T. Still U., School of Osteopathic Medicine*	5	4	5	4	5	5	4	32
Total	19	16	19	15	16	17	12	113

Abbreviations: AHC, Academic health center; COM, College of Medicine; GME, graduate medical education; LCME, Liaison Committee on Medical Education; UME, undergraduate medical education.

### 3.3 Survey instrument

We developed a survey instrument for the purpose of this study (Data S2). The research team collaborated on item development, followed by eight cycles of edits, to ensure alignment with the research question. The survey was pilot tested with five faculty members and two students, resulting in several modifications. The survey was distributed and data were collected through REDCap (Research Electronic Data Capture) hosted at PSCOM.^21^

### 3.4 Study participants and data collection

An investigator at each AHC invited participants in seven pre‐identified groups representing a broad sampling of healthcare professionals (Data S3): (a) medical educators, (b) researchers, (c) health system leaders, (d) hospital administrators, (e) clinical providers, (f) resident/fellow physicians, and (g) health professions students. Investigators were encouraged to use their unique context insights and professional relationships to select participants who could provide thoughtful responses. Lead investigators sent an e‐mail invitation with a survey link to five participants from each category (n = 35 at each AHC; n = 175 total); they were encouraged to invite participants from different roles and training levels within each category. For example, in the resident physician category, different specialty areas were considered. Reminder emails were sent to all participants weekly for 4 to 6 weeks. No incentives were offered.

### 3.5 Data analysis

We used an exploratory qualitative design and thematic analysis approach, allowing us to enhance our understanding of research aims that have been less well addressed in the literature.^17^, ^18^, ^22^ During data analysis, we identified our biases, specifically that this study was developed and performed primarily by educators, researchers, and physicians.^23^ To address this potential bias and ensure confirmability of our results, we asked survey questions in a neutral manner, and the data analysis appropriately balanced all missions.^24^ We included several crosschecks of data with research team members.

Two investigators (J.G., M.D.) used constant comparative analyses to jointly code several survey responses to generate a preliminary codebook to facilitate subsequent analysis.^17^ They then analyzed half of the data, with regular adjudication sessions, to compare codes for inconsistency and agreement; the codebook was updated/modified as necessary. The remaining transcripts were analyzed independently, followed by regular adjudication sessions. Investigators discussed the emergence of higher‐order themes, and further articulated the themes through discussions with co‐investigators. We anticipated strategies would be identified that linked any two of three missions. To capture these results, we were sensitized to the concept of knowledge flows, or practices that result in acquisition of knowledge. Used in prior work related to aligning academic missions in Europe, knowledge flows encourage practices to promote, nurture, and align different areas.^16^ In addition, based upon our prior and current work, we were open to categorizing the results within the health systems science (HSS) framework (if applicable), which is defined as the “principles, methods, and practice of improving quality, outcomes, and costs of healthcare delivery for patients and populations within systems of medical care.” The HSS framework originated within education but has been advanced as critical for integrating research and operations.^25^, ^26^, ^27^, ^28^, ^29^ We anticipated that several areas of synergy between the missions would be related to HSS concepts. Analysis was performed with data management support from the program Atlas.ti 6.0 (Scientific Software, Berlin, Germany).^30^, ^31^ The research team reviewed and agreed upon results.

## 4 RESULTS

Of the 175 participants invited, 113 completed the survey (response rate 65%, Table 1); response rates ranged from 48% (students) to 76% (educators, system leaders). Ninety pages of double‐spaced text data were analyzed. We identified three results categories. First, five higher‐order themes emerged related to aligning all missions. Second, strategies for each mission to better align with the organizational direction were identified. Third, methods for integrating each dyadic pair of the missions, including research‐education, education‐clinical operations, and clinical operations‐research were identified. Representative quotations are provided for each section.

### 4.1 Higher‐order themes

**FIGURE 1:**
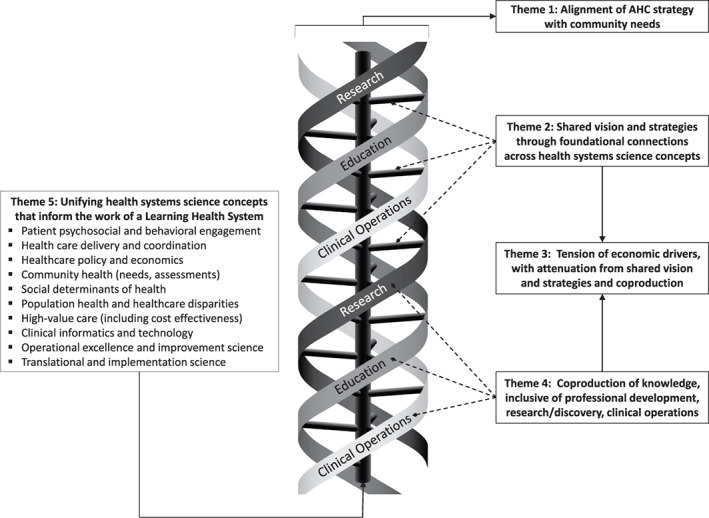
The Evolving Triple Helix of Academic Health Center Tripartite Missions. The figure shows the three academic missions—education, clinical operations, and research—cohesively unified in a triple helix formation in relation to the five higher‐order themes identified in the study. The work of the academic missions needs to be aligned with community needs (Theme 1). The shared vision and strategies (Theme 2) and coproduction of knowledge (Theme 4) occur across all three missions. These are subject to, and can attenuate, the tension of economic drivers (Theme 3). The linking components of the triple helix are held together by the unifying health systems science (HSS) concepts (Theme 5)

We identified five higher‐order themes that spanned all missions. These themes were not specific to any one mission, but rather highlighted the interconnected nature of each. These features are considered ideal and aspirational in the process of fully aligning AHC missions. Figure 1 depicts the relationship between all five themes.

1. **Shared vision and strategies**. Many participants identified the need for a shared vision for the AHCs' work. Lack of a shared vision attenuated the work of each mission area. In addition, some highlighted that when a vision is created, full alignment occurs only when the process is transparent and shared among employees and community stakeholders.

- *There needs to be less siloing of various missions and a more holistic view of the (often competing) education/research/care trilogy. Institutional goals need to be clear about how those things intersect. Otherwise, it will tear itself apart as different factions fight for money, personnel,* etc. [clinical provider]
- *Given clinical care will continue to provide support for education and research, it's important for clinical operations to help develop coherent platforms to organize education of future healthcare professionals as team members, and developing research programs that are relevant to investigators and help move performance forward*. [administrator]
- **Alignment of AHC strategy with community needs**. Many participants believed AHC alignment with community needs and values was critical for success, and could be improved. The AHC's goals should include a larger focus on community needs rather than financial security.
- *[We need to] immerse learners in value‐based care, helping them learn interventions to change at the provider, practice team, division/department, hospital/group, and system levels. Learning how to engage patients in this journey is critical. Linking with community resources will help close the loop*. [administrator]
- *Clinical care in academia needs to more closely align with community needs and should be the model for the community as opposed to an outlier*. [educator]
- **Tension of economic drivers**. Participants identified a tension between the three missions, with clinical operations seen as providing financial support for research and education. In particular, the education mission was often viewed as vulnerable, despite its potential to be a differentiator among competitor health systems. Some respondents described the tension and uncertainty of seeking clinical revenues to support educational innovation. In addition, some believed financial drivers are in conflict with the needs of patients and communities.

- *Even though there is a pressure to prioritize service over education, clinical leadership needs to embrace education as a core mission and make efforts to balance service and education*. [system leader]
- *There will always be tension among clinical, educational, and research missions. Success in education and research will inevitably mean some degree of compromise of the clinical mission*. [administrator]
- *The relative value unit (RVU) cannot rule all. [There needs to be] allowance for specialization. Some people will be more research/education inclined, or clinically‐focused. [There needs to be] balance within a department to achieve all missions*. [resident/fellow]
- **Coproduction to become a learning health system**. One unifying theme was the need for improving structures and work processes to allow for knowledge, vision, values, and culture to be mutually developed between stakeholders. This “coproduction” of work was believed to be a better method to achieve long‐term goals and patient outcomes, and ideally occurs through collaborative work, inclusive of leadership, clinicians, and patients. Within this process, participants frequently cited the need to form new or strengthen already‐existing relationships with other stakeholders, across missions and outside of AHCs. These partnerships include community organizations, clinical sites and hospitals, and patients.

- *We need to shift to the framework of learning health system. This will empower all professionals to be part of the solution ‐ clinicians inform researchers, who inform clinical/education*. [researcher]
- *Health systems are underperforming in their missions. It's not because of bad missions, but rather bad vision and strategies. There's a “values dissonance” within senior executives, mid‐level management and staff, which fosters distrust. There needs to be “disruptive innovation” within the “C‐suite,” and become a member of the clinical team. The model of Dean, CEO, board, chairs, chiefs is antiquated. Health care has outgrown the structure, processes, culture, and incentives utilized by AHCs. The organizational chart needs to be critiqued to foster interdependency between administrators and providers. The strength of doctors and nurses comes from supportive and understanding systems, and the strength of the system is its providers*. [system leader]
- *[AHCs need to] help the public understand when it is appropriate to access and utilize various levels of care. How do we get the public to use the Emergency Department for emergencies? When will insurance companies work for the common good rather than their bottom line? Many solutions are bigger than medical schools and require conversations outside of medicine*. [educator]
- *I see healthcare getting back to its mission of putting providers in position to help people. Burnout exists because non‐profits are being run like “for profits.” For‐profits are designed to “make money” and satisfy stockholders. Non‐profits have a multiplicity of customers (patients, doctors, staff, insurance, pharma) most of whom are not satisfied, especially doctors and nurses, since they have been surgically excised from the value stream*. [system leader]
- **Unifying systems concepts at the nexus of the three missions**. Participants identified a mutually shared set of unifying systems concepts within each mission, suggesting all three missions should be increasing focus on these concepts (eg, social determinants of health) for alignment and improving outcomes. These unifying concepts align with the principles included in the HSS framework.

- *[We need] more training in interprofessional education, technology, social determinants of health, behavioral medicine, teamwork, and motivating patients*. [researcher]
- *Large data sets will shed light on waste, and pinpoint inappropriate spending, which will change practice. Electronic medical records will make it possible for provider feedback to become an instrumental part of education, and “big data” will make cost‐conscious care reality*. [student]
- *I hope we see more funding for research and quality improvement that focuses on waste, stewardship of resources, variation in care, polypharmacy, and patient self‐management*. [administrator]

### 4.2 Mission‐related strategies

For each mission area, participants identified strategies that would better align each mission with AHC goals. Table 2 shows the categories and strategies for each area. Several of these strategies were identified by participants as already underway in AHCs, or need to be pursued in the coming years.

**TABLE 2:** Education, research, and clinical strategies for improving academic medical center alignment

Mission area	Category	Representative areas and examples
Education	Competencies/curricula	Increase focus on health systems concepts and chronic disease management (Figure 1) Develop higher‐order competencies (eg, inquiry, clinical reasoning) Cultivate life‐long learning
	Instructional methods	Accelerate competency‐based medical education and evaluations Increase early clinical exposure (less focus on classroom knowledge transfer) Enhance integration of curricula to decrease isolation of learning concepts
	Recruitment and development of evolving workforce	Recruit individuals with diverse backgrounds Enhance admission efforts to recruit individuals seeking to work with vulnerable patients Incentivize faculty to stay within the system at point of recruitment, and beyond Implement accelerated programs to decrease time spent in specialty‐specific training Promote faculty development to improve knowledge and skills
Research	Shifting research agenda	Emphasize needs of patients, communities, and systems (Box 1) Enhance research and evaluation of systems needs Enhance research and evaluation related to medical education
	Developing partnerships and researchers	Establish relationships with stakeholders within AHC Leverage clinical sites with expanding clinical networks and systems Collaborate with community‐based partners and patients Recruit researchers to align with new mission focus Include diverse stakeholders from across professions in new partnerships
	Operations	Enhance accessibility of researchers and data for larger AHC Promote cross‐disciplinary functions Enhance transparency of data and work Focus scholarship and increased work outputs based on evolving mission Enhance education for researchers
Clinical (aligned with concepts in Figure 1)	Care delivery models and technology	Incorporate new team‐based models and increase interprofessional collaboration in care Implement integrated and standardized care pathways and protocols Allow clinicians to perform tasks within these models commensurate with skillset Integrate information technology (eg, web applications, portals) within care processes Streamline information exchange between different electronic health record platforms Incorporate telemedicine and telehealth to provide care for underserved patients Use of artificial intelligence to improve care processes
	Focus on patient factors and needs	Address social determinants of health Address chronic disease management and prevention Improve behavioral and mental health services
	Value‐based care	Shift payment structure to value to improve health of population of patients Increase cost transparency for both clinicians and patients Improve data transparency to drive the shift to value Ensure standardization across all care settings (eg, inpatient, outpatient, etc.) Incentivize practice for value‐based care
	Clinician well‐being	Focus on well‐being among clinicians to ensure long‐term viability of health system Address burnout mediators and facilitators using a system‐wide approach

#### 4.2.1 Education

Participants identified diverse areas to focus on education to improve alignment with AHC goals. Categories included: (a) new curricula aligned with system needs, (b) different pedagogies, and (c) increased focus on recruitment and development of an evolving workforce.

New curricula:

- *Incorporation of high‐value care, patient safety, population health into curriculum will be important. People in AHCs have a tendency to avoid business aspects of medicine, but business is a reality that people cannot avoid*. [system leader]

Educational strategies:

- *Continuously challenge students to think harder, faster and better by exposing them to patients, encourage team‐based education, ensure trainees are the primary focus rather than provider productivity, and incorporate students into research/ quality improvement for early exposure*. [resident/fellow]
- *Curricular changes supporting students' ability to become self‐authoring and adaptive learners. This will require instructional design change that moves away from classrooms and into clinical learning environments. Essential for professional development efforts, faculty need to be provided with effective, frequent, timely feedback on their abilities*. [medical educator]

Recruitment and development of evolving workforce:

- *[Education needs to] look at admissions policies, stick to guidelines that align with the mission. Do not deviate. Remind students of the mission throughout the curriculum with activities and learning*. [clinical health system leader]

#### 4.2.2 Research

Participants identified several categories of strategies to align research with AHC goals. Categories included: (a) evolving research agenda that includes not only basic science and “discovery,” but also patient‐centered care and “delivery” research (Figure 1), (b) developing partnerships and researchers, and (c) operations.

Shifting research agenda:

- *There will always be the need for basic science research ‐ only the subject matter will change. Growth in primary care research needs to be encouraged because too much of what we do is based on hearsay, “that's the way we do things.” Research needs to expand horizons to include studies on social determinants of health that impact population health*. [clinical provider]

Developing partnerships and researchers:

- *[Research should] focus more on embedded research within health systems so results are meaningful across diverse stakeholders rather than research that is interesting only to a set of “like‐minded” researchers*. [researcher]
- *Before starting research, understand system needs regarding the topic, by bringing in focus groups of all system participants: social workers, physicians, patients, staff*. [student]
- *Provide training opportunities for fellows, students, faculty. Develop an institutional budget that provides time for physicians to do research. Provide mentoring for grant development. Recruit physicians with funding and robust programs, and strong clinical presence*. [researcher]

Operations:

- *As grant funding is expected to decrease, research programs need to figure out ways to conduct research that does not require large funding, apply “lean” [management] to research programs, promote interdisciplinary team‐based research to share costs*. [system leader]
- *Having a clear understanding of the mission up‐front is important. Often research happens in a vacuum. Having a uniting focus/mission will allow programs to better align their studies with institution and community goals*. [student]

#### 4.2.3 Clinical

Participants identified several strategies where clinical operations could better facilitate alignment with AHC goals. Categories included: (a) new delivery models embracing team‐based care, standardized care processes, and technologies, such as web‐based applications and telemedicine, (b) need to focus on patients' social influences on health, (c) shift to value‐based care, inclusive of payment structure, incentivizing physicians, and increased data transparency, and (d) clinician well‐being.

New care delivery models and technology:

- *Build functional TEAM units that provide transdisciplinary care, identify social disparities, and develop systems to assist and connect patients when needed*. [system leader]
- *[Clinical operations need to be] more integrated, team‐driven, patient‐centered, outcome‐driven, less hospital‐centric, and highly reliable by 2027*. [administrator]

Patient factors and needs:

- *Focus on community‐based care and prevention though healthcare positions that support optimization of social determinants of health*. [medical educator]

Value‐based care:

- *There are efforts in every clinic to make care more efficient, accessible, and higher quality. However, efforts are disconnected ‐ bringing stakeholders together to align missions and avoid duplicative work would allow for greater efficiency and impact*. [student]
- *Bring the message of value‐based care to all. Learn how to incorporate data to understand how we are doing in quality, patient satisfaction, cost and utilization and provider/care team satisfaction*. [administrator]

Clinician well‐being:

- *Clinical operations need to care about outcomes other than the bottom line, and also show they actually care about patients and hardworking providers/staff*. [researcher]

### 4.3 Collaborative knowledge flows between missions

Participants identified knowledge flows that demonstrate how one mission can directly inform or influence the growth of another mission, which then indirectly influences the third mission. Findings within each of the three pre‐identified dyadic pairs include: (a) research‐education—including research on education, shared resources, and integration of faculty and students within research‐based work, (b) clinical operations education—including content and competencies, methods and evaluation, clinical learning environment, recruitment, and professional development, (c) research‐clinical operations—including clinically relevant research agenda and new collaborations and organizational structures/processes that promote aligned work. Table 3 describes these areas, with themes and quotations for each.

**TABLE 3:** Potential categories of enablers to enhance alignment between the research, education, and clinical missions

Dyad	Category	Description and representative quotation
*Research‐Education*	Research on education	Focus on evaluation and outcomes of traditional and new education methods and innovations. *More research about what makes a competent doctor would determine if it supports our theory about the approach to training doctors. So much research is focused on academic outcomes and board scores that we are forced to move away from our primary mission to achieve those goals instead. [clinical health system leader]* *As new educational methods and strategies are implemented, research can be directed at evaluating relative effectiveness of different strategies. A standard expectation for these programs to provide ongoing evidence is needed. [clinical health system leader]* *Increase opportunities for grant‐funded education research. Leverage high‐quality clinical simulation and simulation‐modeling research. Provide trainees with opportunities to immerse in the clinical domain to start framing research questions early in their training. [medical educator]*
	Shared resources	Streamline and share common resources among educators and researchers (eg, library resources, education programs). *Multidisciplinary research would allow less research to be done with more people being involved. Medical students, nursing students, nurses, and physicians are doing their own research when a team approach could be utilized to find the best outcome for patients and have a cohesive research result. [clinical provider]*
	Integration of faculty and students with research‐based work	Inclusion of faculty from both education and research and health professions students in integrated activities and programs. *Encouraging all levels of trainees to get involved with research will spread the need for improvement and teach trainees how to do this at an earlier stage. [student]* *Research programs should adopt longitudinal, multi‐year studies led by residents but include interprofessional students. [medical educator]* *Better alignment of translational research with education could better integrate faculty, which would require substantial culture change, since these two missions are far apart. The relative paucity of physicians carrying out research leads to divergence and lack of understanding/ respect for the missions. [researcher]*
*Education‐Clinical*	Content and competencies	Focus on health systems science areas in both education and clinical care transformation (Figure 1). *In recognition of the continuing changes in healthcare delivery and financial pressures on academic medical centers, health professions education should focus on systems of care, interdisciplinary education, and ways to improve health status of entire communities. [hospital/health system administrator]*
	Methods and evaluation aligned with clinical needs	Reflect behaviors occurring in clinical environments related to care delivery (eg, curiosity, systems thinking, humility) that should also add value to the system. *Clinical leaders should be more involved in educating trainees who they hope to bring into their clinical enterprise after graduation. [clinical health system leader]* *The more care can be integrated with basic science in medical school, the more both components will be understood. The more physicians and scientists interact and discuss teaching approaches, the more the students will benefit. [researcher]* *Increase resident supervision/pay, decrease responsibility to match abilities. Residents should not be used as reduced‐cost labor. [hospital/health system administrator]*
	Clinical learning environment redesign	Redesign and improvement of care models and processes that support education in systems learning areas (Figure 1). *Relax the time limits that providers have with their patients, particularly if those providers are also training students.[resident/fellow trainee]* *Ensuring there is enough faculty and staff for each specialty to provide care for patients, but also to allow enough time for extra medical education.[student]*
	Recruitment and professional development	Recruit faculty with skills/mindset and enhance the skill of current faculty to both transform care environments and improve education in systems areas. *Added training of all clinical preceptors we work with so they understand the importance of our mission and do not unintentionally detract from that. Provide a certificate of added qualification to preceptors who agree to go through added training to enhance their abilities as an educator. [clinical health system leader]*
*Clinical‐Research*	Clinically relevant research agenda	Focus on care delivery and innovation (Figure 1). *Research programs that help health systems determine ways to improve operational effectiveness, empower patients to be more responsible and impact their own health, and improve outcomes of care will be better aligned with AHC clinical missions. [hospital/health system administrator]* *Collect data and contribute to research on clinic flow, efficacy in scheduling, treating patients, maintaining patient communication. [resident/fellow trainee]* *There should be an increase of QI research, focusing on healthcare spending/utilization to help deliver appropriate care and reduce costs. [student]* *If population health or care redesign is a goal, it will need guidance from research, so those goals are aligned. [Right now], they are completely separate. [researcher]*
	Collaborative organizational structures and processes	Create new or evolve organizational structures and processes that bring together interprofessional expertise (eg, researchers, clinicians, informatics, engineers, and clinical leaders) to facilitate more assimilated research. *With the introduction of the Clinical and Translational Science Institute, our institution has helped to better align our research mission, promoting clinical/translational research, which will ultimately improve public health. [researcher]* *Talking together is important. The best research will be done with interprofessional teams and patients to examine care from a team perspective. [medical educator]*

## 5 DISCUSSION

A century ago, academic health centers (AHCs) emerged at the intersection of patient care, research, and education. These tripartite missions were largely embodied in people, specifically faculty known as “triple threats” for their breadth and depth of expertise in all three areas.^32^, ^33^ AHCs drew their identities from these individuals, and, in turn, supported their efforts. Medicine has changed significantly over this time period—“triple threats” are rare, and, in their absence, the previously coherent AHC identity linked to these individuals has unraveled.^34^ Each mission area has become more demanding, and most faculty members operate mostly within one or two missions.^32^ We can no longer depend on individual people alone to define the AHC identity—institutions need to take the lead and support the diverse individuals who can make it work. While each mission is independently important, the unique potential of the AHC to fulfill its promise in achieving the Quadruple Aim and becoming a learning health system lies in fortifying the intersection of all three missions.^2^, ^5^ This will require: (a) understanding of the perspectives of the people who comprise AHCs, (b) insight into opportunities for progress and transformation in effective mission alignment, and (c) deliberate investment by AHCs in the resources and infrastructure necessary to facilitate the learning health system journey. This study was designed to explore the voices of AHC stakeholders, and has provided insight into a way forward. Our participants articulate how each mission's strategies may inform the others in a more cohesive manner, specifically through shared vision, alignment, coproduction, and mutually shared systems concepts.

Prior work has explored alignment across missions, highlighting the need to account for economic, management, governance, and strategy considerations.^35^ These works have been primarily conceptual, or propose granular strategies within research and clinical missions (eg, population health), or education and clinical missions (eg, workforce gaps).^14^, ^36^, ^37^, ^38^, ^39^, ^40^, ^41^, ^42^, ^43^, ^44^, ^45^, ^46^, ^47^, ^48^, ^49^, ^50^ Missing from this work has been the perspectives of system leaders, clinicians, scientists, and trainees who increasingly dedicate their professional careers to one mission. Uncovering these perspectives provides a fresh lens through which to visualize and address this challenge and create environments in which stakeholder voices facilitate systems level changes toward a common goal.

In the past two decades, the concept of a learning health system has emerged to conceptualize the organizational structures and processes for leveraging the iterative use of data and learning to generate knowledge and support evolving systems of care.^3^, ^4^, ^5^ A learning health system uses rapid cycles to identify problems, draw on stakeholders contributions to ensure alignment with perceived needs, implement small‐scale innovations, use evaluation and timely feedback, apply objective evidence to improve care, and pursue open dialogue with stakeholders to reinforce a learning culture. Our participants advance the concept of the learning health system by identifying two key issues that can help operationalize the concept, particularly within AHCs: (a) coproduction as a guiding principle of process and strategy, and (b) health systems concepts as foundational content for evolving the AHC identity. Coproduction, or the meaningful collaboration among stakeholders in planning, implementation, and evaluation, was identified as currently missing from effective work across missions.^51^, ^52^ While used “vertically” within the context of healthcare delivery and redesign, our results argue for coproduction to be applied “horizontally” across missions, supporting the uncovering and evolution of a unique AHC identity.^51^ Educators, researchers, and system leaders need to be equally contributing as colleagues—sharing expertise, respect, and professional investment—rather than operating from distance and encumbered by power differentials, different languages and priorities, and unaligned outcomes. If each mission continues to stay in its own lane—education in content/pedagogy, research in grants and publications, and clinical care in unaligned system initiatives—then the opportunity for AHCs to realize their unique identity as change agents capable of improving outcomes will continue to be compromised.

The second major issue relates to unifying concepts that inform the substrate of coproduction. These concepts (Figure 1) overlap with the HSS framework, which originated within medical education but has been proposed as a unifying framework for advancing research and clinical operations.^25^, ^26^, ^27^, ^28^, ^29^ HSS has the depth and reach to support coproduction across missions. Some authors have suggested more limited applications, such as a recent insightful commentary suggesting AHCs consider adding a fourth mission to the tripartite model focusing on social accountability.^39^ Rather than adding a new mission, our results argue for using HSS as a conceptual framework and substrate for creating AHC strategic alignment.

The health of AHCs depends on a vibrant and strategically sound identity cultivated by a coproduction process anchored in HSS. This identity, once shaped by the careers of individual “triple threats,” needs to be deliberately reimagined and woven from a mutually supportive, institutional collaborative triple helix of clinical care, education, and research (Figure 1). The *raison d'être* for AHCs has not changed, but the strategies for ensuring their health and sustainability have. This study suggests a new focus for an AHC identity, but not an exclusionary one. Research and education in non‐HSS areas absolutely need to continue. But the future will depend, in part, on a mutually shared mental model that requires an evolution of the research and education agendas.

Our participants suggested the development of new centers or institutes to accomplish a co‐produced, HSS‐centric agenda. Seeking to raise the profile of AHCs and improve health, the work of building these centers has already begun as demonstrated in the Dartmouth Institute for Health Policy and Clinical Practice, the Mayo Clinic Kern Center for the Science of Health Care Delivery, and Department of Population Health/Division of Healthcare Delivery at New York University School of Medicine.^45^, ^50^, ^53^, ^54^, ^55^, ^56^ These new centers are evidence of an already evolving AHC identity, and a pragmatic strategy that encapsulates core tenets of a learning health system, such as the identification of key challenges, stakeholder inclusion and collaboration, and iterative use of data to inform healthcare redesign.^3^, ^4^

There are several limitations to this work. First, data were obtained from open‐ended surveys sent to participants, which may limit the richness of data. However, we received a good response rate, and quantity of data was significant. Second, our five medical schools represent a small fraction of U.S. medical schools. We did sample across different medical school‐health system partnerships, which increases generalizability. However, different types of medical schools, teaching hospitals, and faculty practice‐group affiliations exist, and these may not have been well represented.^9^ Future work could be undertaken to re‐examine these findings, with particular attention to the type of medical school‐health system affiliation. Despite these limitations, we believe these results contribute to the literature related to evolving AHC missions, and provide a foundation for subsequent scholarly work.

AHCs are at a crossroads with respect to identity and alignment across the education, research, and clinical missions. The AHC identity is at risk of being co‐opted by clinical operations, leaving unaligned research and education to survive on trickle‐down and external funding. The voices in this study argue for research and education to join health systems as full, co‐producing partners in fulfilling the AHC vision of improving the health of patients and populations. This is the challenge, and the enormous opportunity, of these unique institutions.

## CONFLICT OF INTEREST

To our knowledge, no conflict of interest, financial or other, exists for all authors. The views expressed in this paper reflect the views of the authors and do not necessarily represent the views of the AMA, the Josiah Macy Jr. Foundation, or other participants contributing to this work. Dr. Gonzalo is co‐editor of a textbook entitled Health Systems Science (Elsevier, 2016 and 2020) and co‐editor of the textbook entitled Health Systems Science Review (Elsevier, 2019). There are no other conflicts of interest to report.

## Supporting information


**Data S1.** Categories of medical schools and health systems relationships used in research study

**Data S2.** Participant survey

**Data S3.** Categories of study participants
