# Supplementary material for: The triple helix of clinical, research, and education missions in academic health centers: A qualitative study of diverse stakeholder perspectives
Source: Learn Health Syst. 2020 Oct 17;5(4):e10250. doi: 10.1002/lrh2.10250 (PMC8512738; doi:10.1002/lrh2.10250)
Supplement: Supplementary file 1 — Data S1. Categories of medical schools and health systems relationships used in research study [file LRH2-5-e10250-s003.docx]

**Appendix 1: Categories of Medical Schools and Health Systems Relationships Used in Research Study**

The following four categories of medical schools and health system relationships were used in this research study. At least one medical school from each category was identified and included in the study. (Abbreviations: AHC=academic health center; COCA= commission on osteopathic college accreditation COM=college of medicine; GME=graduate medical education; LCME=liaison committee for medical education; UME=undergraduate medical education)

1. Integrated AHC with COM in a public comprehensive or health science university
2. Integrated AHC with COM in a private comprehensive/health science university
3. LCME- or COCA-accredited COM in a public comprehensive/health science university with affiliation agreement (not under common ownership) with ≥1 AHC that sponsors and significantly participates in undergraduate medical education (UME) and graduate medical education (GME)
4. LCME- or COCA-accredited COM in a private comprehensive/health science university with affiliation agreement (and not under common ownership) with ≥1 AHC that sponsors and significantly participates in UME and GME.
