# Supplementary material for: The triple helix of clinical, research, and education missions in academic health centers: A qualitative study of diverse stakeholder perspectives
Source: Learn Health Syst. 2020 Oct 17;5(4):e10250. doi: 10.1002/lrh2.10250 (PMC8512738; doi:10.1002/lrh2.10250)
Supplement: Supplementary file 2 — Data S2. Participant survey [file LRH2-5-e10250-s002.docx]

**Appendix 2 - Participant Survey**

**Background of study to be included at the start of survey:**

Background of Study: In this study, we are exploring the present and future role of medical schools within Academic Medical Centers (hospital affiliated with a medical school and/or a residency training program). The study will explore how educators, researchers, administrators, health care professionals, and trainees believe the academic mission is shifting, and ways in which medical schools and hospitals can better align. The questions below relate to your perspective regarding education, research, and clinical care, and what strategies can be used by other institutions to better align the education, research, and clinical missions. Please answer these questions from your perspective as a health care professional, educator, researcher, or trainee. The data will be anonymous, and will not be linked to any one role/title.

**Items**

1. Please identify the primary category of your job role in the health system:
   1. Educator
      1. Education leadership role: _____
      2. Undergraduate medical education
      3. Graduate medical education
      4. Continuing medical education
      5. Other health professions education
   2. Researcher
      1. Research leadership role: _____
      2. Basic science or “bench” researcher
      3. Clinical science researcher
      4. Researcher - other
   3. Clinical health system leader
      1. Department chair
      2. Vice chair
      3. Division chief
      4. Director of program or group
   4. Hospital/health system administrator
   5. Clinical provider
      1. Nurse providing care within the health system
      2. Mid-level provider (e.g. nurse practitioner, physician assistant) providing care within the health system
      3. Physician providing care within the health system
      4. Other provider
   6. Student
      1. Medical student
      2. Student – other health professions program
   7. Resident/fellow trainee
      1. Resident physician
      2. Fellow physician
      3. Other trainee

2. Please list all of your job title(s) and role(s) - e.g. Vice Dean for Research, Sr. Associate Dean, Fellow Physician in Infectious Disease etc.: ____________

1. How many years have you been employed in a health care environment affiliated with a medical school? ___

(for those responding as a “student/resident/fellow trainee” – In what year of training are you currently in? e.g. PGY1, MS1, etc._____)

4. Changes in health care, education, and research:

1. How do you see health care delivery changing over the next 10 years?
2. Do you think health care delivery needs to change? Please explain why or why not.
3. How do you see health professions education or research changing over the next 10 years?
4. Do you think health professions education or research needs to change? Please explain why or why not.
5. How do you see the role of health professions students within academic medical centers changing over the next 10 years?
6. Do you think the role of health professions students within academic medical centers needs to change? Please explain why or why not.

5. Missions of academic medical centers:

- 1. Will these changes in health care affect the clinical, education or research missions of academic medical centers? Please explain why or why not.
  2. What specific strategies can health professions education programs adopt (or have already adopted) to better align all missions?
  3. What specific strategies can research programs adopt (or have already adopted) to better align all missions?
  4. What specific strategies can clinical care and operations adopt (or have already adopted) to better align all missions?

6. Please provide any additional comments particularly as they relate to your views on how academic medical centers should adapt to changes in health care, the future role of academic medical centers, or other related ideas.
