# Supplementary material for: The triple helix of clinical, research, and education missions in academic health centers: A qualitative study of diverse stakeholder perspectives
Source: Learn Health Syst. 2020 Oct 17;5(4):e10250. doi: 10.1002/lrh2.10250 (PMC8512738; doi:10.1002/lrh2.10250)
Supplement: Supplementary file 3 — Data S3. Categories of study participants [file LRH2-5-e10250-s001.docx]

**Appendix 3: Categories of Study Participants**

Each co-investigator was asked to identify potential participants from the following categories and characteristics:

1. Educators (n=5)

Consider inviting participants from the following categories/roles:

- 1. Leadership role overseeing educational mission. Potential roles could include Vice Dean for Educational Affairs, Associate Dean or equivalent.
  2. If applicable, health professions leadership role, i.e. nursing school dean, graduate program.
  3. Educator in undergraduate medical education
  4. Educator in graduate medical education
  5. Educator in continuing medical education/faculty development

1. Researchers (n=5)

Consider inviting participants from the following categories, and individuals who may fit the following categories and also be educators within the medical school, and/or those who are physician scientists, etc.:

1. Leadership role overseeing the research mission. Potential roles could include Vice Dean for Research, Associate Dean or Equivalent.
2. Researcher(s) with substantial research funding in basic science area (e.g. PhD investigators)
3. Researcher(s) with substantial research funding in clinically-based research
4. Clinically-based health system leaders (n=5)

Consider inviting participants from the following categories:

- 1. Clinical department chairs (or Vice Chair for Quality, etc.)
  2. Division chief(s)
  3. Director of clinical program (i.e. Hospitalist Director, Ambulatory Service Director).

1. Hospital/health system administrators (n=5)

Consider inviting participants from the following titles or roles (non-inclusive list):

- 1. Dean/Chief Executive Office of Health System
  2. Chief Quality Officer, Chief Nursing Officer, Chief Medical Officer

1. Clinical providers (n=5)

Consider inviting participants from the following titles or roles (non-inclusive list) - these participants should preferentially not spend the majority of their time in education, research, or in a health system leadership role, but represent the frontline of the health system.

- 1. Nurse providing care within the health system
  2. Mid-level provider (e.g. nurse practitioner, physician assistant) providing care within the health system
  3. Physician providing care within the health system

1. Student learners (n=5)

Consider inviting participants from the following titles or roles (non-inclusive list)

- 1. Medical students
  2. Other health professions students (e.g. nursing, physician’s assistant, or pharmacy).

1. Resident and fellow physician trainees (n=5)

Consider inviting participants from the following groups (non-inclusive list) – with preference for diverse representation from specialties:

- 1. Resident physicians
  2. Fellow physicians
  3. Other trainees
